# Evaluation of input geological parameters and tunnel strain for strain-softening rock mass based on GSI

**DOI:** 10.1038/s41598-022-23587-x

**Published:** 2022-11-29

**Authors:** Lan Cui, Qian Sheng, Jun Zhang, You-kou Dong, Zhen-shan Guo

**Affiliations:** 1 State Key Laboratory of Geomechanics and Geotechnical Engineering, Institute of Rock and Soil Mechanics, Chinese Academy of Sciences, Wuhan, 430071 China; 2 University of Chinese Academy of Sciences, Beijing, 100049 China; 3 Key Laboratory of Highway Construction and Maintenance Technology in Loess Region of Ministry of Transport, Shanxi Transportation Technology Research & Development Co., Ltd., Taiyuan, 030032 China; 4 College of Marine Science and Technology, China University of Geosciences, Wuhan, 430074 China

**Keywords:** Civil engineering, Solid Earth sciences

## Abstract

The regression analysis method is being widely adopted to analyse the tunnel strain, most of which ignore the strain-softening effect of the rock mass and fail to consider the influence of support pressure, initial stress state, and rock mass strength classification in one fitting equation. This study aims to overcome these deficiencies with a regression model used to estimate the tunnel strain. A group of geological strength indexes (GSI) are configured to quantify the input strength parameters and deformation moduli for the rock mass with a quality ranging from poor to excellent. A specific semi-analytical procedure is developed to calculate the tunnel strain around a circular opening, which is validated by comparison with those using existing methods. A nonlinear regression model is then established to analyse the obtained tunnel strain, combining twelve fitting equations to relate the tunnel strain and the factors including the support pressure, GSI, initial stress state, and critical softening parameter. Particularly, three equations are for the estimation of the critical tunnel strain, critical support pressure, and tunnel strain under elastic behaviour, respectively; and the other nine equations are for the tunnel strain with different strain-softening behaviours. The relative significance between the GSI, the initial stress and the support pressure on the tunnel strain is assessed.

## Introduction

The tunnel closure should be predicted appropriately as it is utilised to determine the stability of the rock mass and has been adopted in the engineering practices to guide the preliminary support design. Many analytical and numerical methods were proposed to assess the ground reaction curve with different failure criteria, flow rules, and failure behaviours of the rock mass^1–6^. The solutions reveal the relationship between the tunnel strain and the support pressure, which are efficacious for determining the support type with a particular geological condition. However, many solutions are often too cumbersome for practical applications due to its complicated derivation, equations, and multiple geological parameters. In this aspect, empirical methods seem to be more accessible to the engineering practisers due to their simplicity. Rock mass rating^7–9^, geological strength index^10^, and tunnelling quality index Q^11,12^ are the commonly utilised systems to guide the tunnel design by adequately quantifying the strength and deformation properties of the rock mass. Based on previous case back-analysis with assumed rock mass behaviours, the empirical methods often fail to account for the input geological parameters for a specific case. Thus, the strain redistribution and support performance cannot always be well-estimated by the empirical methods.

The regression analysis method has been adopted by many researchers to evaluate the tunnel strain as it takes advantage of the accuracy of the numerical tools and the convenience of the empirical schemes^13–21^. In the existing studies, great amount of data result was obtained using iterative procedures to analyse the large number of tunnelling cases. Multiple geological parameters for each tunnel case were simplified into a single strength parameter, and the rock mass deformation was quantified artificially as a function of the strength parameter using a nonlinear regression model. Among the studies, the functions enable to obtain the tunnel strain or the plastic zone radius for various tunnel cases with various geological scenarios. However, the limitation is obvious due to the difficulty when considering the strain of rock mass showing strain-softening behaviours, which is proved to be a common behaviour in numerous rock tests^3^. Also, many studies adopted only one fitting equation in the regression model, failing to consider the support pressure, the initial stress, and the strength classification (such as RMR, GSI, and the compressive strength). As a result, the application of analysis results with one fitting equation is limited to particular initial stress or rock mass quality.

In this paper, the index GSI is assigned with a group of values to represent the strength parameters and the deformation moduli for a strain-softening rock mass having various qualities. The tunnel strain around a circular opening under a hydrostatic stress state is obtained through a numerical scheme, which is validated through comparison with the previous studies. A more accurate estimation of the tunnel strain is further derived by semi-analytical procedures with different input geological parameters. Twelve fitting equations are proposed with the regression analysis method to correlate the tunnel strain with the support pressure, the GSI, the initial stress state, and the critical softening parameter; In particular, three equations are for the critical tunnel strain, the critical support pressure, and the tunnel strain in the elastic zone, and nine equations are for the tunnel strain in the plastic zone with different strain-softening behaviours.

## Problem setup

### Assumptions

Some assumptions are considered prior to the analysis:

1. A circular opening, with a radius of *R*_0_, is under a hydrostatic stress field of $\sigma_{0}$ asymmetrically distributed around it; the radial stress $\sigma_{{\text{r}}}$ and the tangential stress $\sigma_{{\uptheta }}$ correspond to the minor and major principal stresses $\sigma_{3}$ and $\sigma_{1}$, respectively;
2. Plane strain condition is considered as the deformation along the longitudinal direction of the tunnel is virtually uniform;
3. Material of the rock mass is isotropic, continuous, and initially elastic. Near underground excavations where confinement is reduced, most rock mass exhibits post-peak strength loss, which is called strain-softening property. The rock mass presents strain-softening (SS) behaviour; the elastic-perfectly-plastic (EPP) and elastic-brittle-plastic (EBP) behaviours are also considered, which are taken as special cases of the SS behaviour. The SS, EPP, and EBP behaviours of the rock mass induced by excavation operations are shown in Fig. 1. A support pressure *p*_i_ is evenly imposed around the tunnel. *σ*_r2_ and *σ*_θ2_ represent the radial and tangential stresses at the elasto-plastic boundary, respectively. Within a SS rock mass, *σ*r1 and *σ*θ1 are the radial and tangential stresses at the plastic softening-residual boundary, respectively. The radii of the plastic softening and residual areas are symbolised as *R*_p_ and *R*_r_, respectively. For the EPP and EBP rock masses, the radius of plastic area is represented as *R*_p_

**Figure 1:**
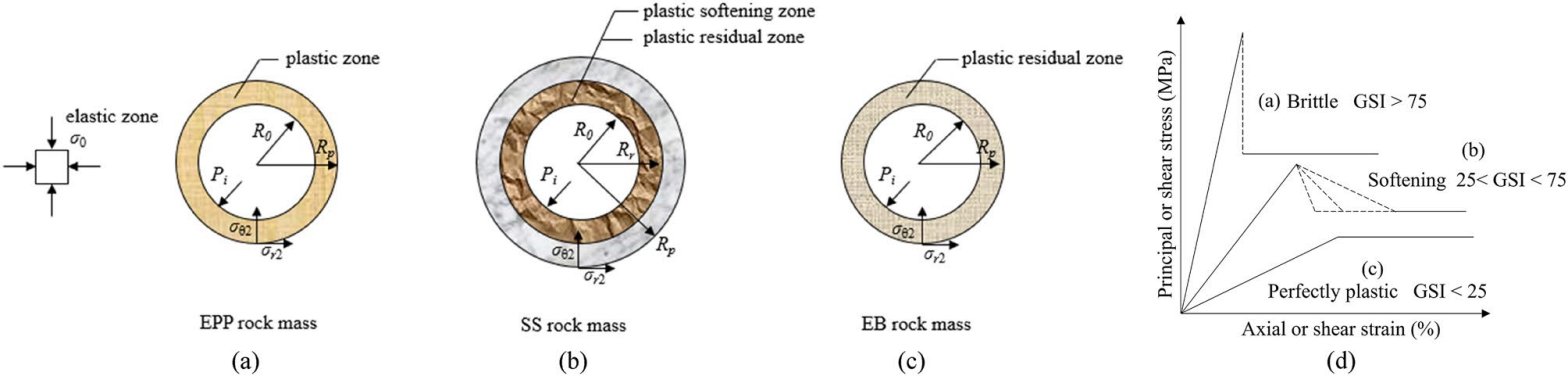
Schematic graph of excavation problem and stress–strain relationship: (**a**) for EPP rock mass; (**b**) for SS rock mass; (**c**) for EBP rock mass; (**d**) stress–strain relationships (reference from^3^).
4. The softening parameter *η* characterises the softening quantity in the rock mass and is calculated as the gap between the tangential and radial plastic strains for the axisymmetric problem:
  1 $$\eta { = }\varepsilon_{{\uptheta }}^{{{\text{plas}}}} - \varepsilon_{{\text{r}}}^{{{\text{plas}}}}$$
  The critical value of *η* is denoted as η^*^, which occurs at the moment that the rock mass strength decays to its residual value. Specially, η^*^ has values of ∞ and 0 for the EPP and EBP rock masses, respectively.
5. The Mohr–Coulomb failure criterion is considered for the plastic potential function^22,23^
  2 $$g\left( {\sigma_{{\text{r}}} ,\sigma_{{\uptheta }} ,\psi } \right) = \sigma_{{\uptheta }} - \frac{1 + \sin \psi }{{1 - \sin \psi }}\sigma_{{\text{r}}}$$
  where *ψ* is the dilatancy angle and herein is taken as nil.
6. The Hoek–Brown (H-B) failure criterion is satisfactory in the quick estimate of the rock mass strength^24^:
  3 $$\sigma_{1} = \sigma_{3} + \sigma_{{{\text{ci}}}} \left( {{{m_{{\text{b}}} \sigma_{3} } \mathord{\left/ {\vphantom {{m_{{\text{b}}} \sigma_{3} } {\sigma_{{{\text{ci}}}} + s}}} \right. \kern-\nulldelimiterspace} {\sigma_{{{\text{ci}}}} + s}}} \right)^{a}$$
  where *σ*_ci_ represents the uniaxial compression strength of the intact rock; *m*_b_, *s* and *a* are strength parameters of the Hoek–Brown rock mass. Because of the axisymmetric condition, the radial stress *σ*_r_ and the tangential stress *σ*_θ_ correspond to the minor and major principal stresses *σ*_3_ and *σ*_1_, respectively. Equation (3) can be transformed as:
  4 $$f\left( {\sigma_{{\text{r}}} ,\sigma_{{\uptheta }} ,\eta } \right) = \sigma_{{\uptheta }} - \sigma_{{\text{r}}} - \sigma_{{{\text{ci}}}} \left( {{{m_{{\text{b}}} \sigma_{{\text{r}}} } \mathord{\left/ {\vphantom {{m_{{\text{b}}} \sigma_{{\text{r}}} } {\sigma_{{{\text{ci}}}} + s}}} \right. \kern-\nulldelimiterspace} {\sigma_{{{\text{ci}}}} + s}}} \right)^{a}$$
  According to the geological observations in the field, Reference^10,24,25^ constructed the relation between the strength parameters (*m*_b_, *s* and *a*) and GSI. The empirical equations are listed as follows:
  5 $$m_{{\text{b}}} = m_{{\text{i}}} \exp \left( {\frac{{{\text{GSI}} - 100}}{28 - 14D}} \right)$$
  6 $$s = \exp \left( {\frac{{{\text{GSI}} - 100}}{9 - 3D}} \right)$$
  7 $$a = \frac{1}{2} + \frac{1}{6}\left( {e^{{{{ - {\text{GSI}}} \mathord{\left/ {\vphantom {{ - {\text{GSI}}} {15}}} \right. \kern-\nulldelimiterspace} {15}}}} - e^{{ - {{20} \mathord{\left/ {\vphantom {{20} 3}} \right. \kern-\nulldelimiterspace} 3}}} } \right)$$
  where *D* is a coefficient influenced by the disturbance from blast impact and the stress relaxation. An optimised blasting operation with an accurate drilling control technique is assumed during the tunnel excavation, thereby, the damage to the tunnel wall is negligible and *D* is regarded as 0 by Hoek^26^. *m*_i_ in Eq. (5) characterises the friction between the composition minerals.

### Strength classification of rock mass

The strength classification systems, such as the RMR, Q, and GSI, were successfully applied to many tunnel excavations. Various empirical equations by the systems are feasible to characterise the strength and deformation behaviours of the rock mass. Herein, GSI is incorporated to quantify the rock mass properties. Advantages of the GSI are demonstrated in three aspects: GSI is directly correlated to the strength constants in the Hoek–Brown failure criterion^24^; GSI can be estimated by RMR and Q systems, thus some strength parameters related to RMR can also be represented by GSI; and the residual strength of the strain-softening rock mass could be calculated from the peak value of GSI based on the equation proposed^27^.

#### Correlation between RMR and GSI

In the latest version, the relationship between GSI and RMR is:

8 $${\text{GSI = RMR}} - 5{\text{, RMR > 23}}$$

It is noted that Eq. (8) is specialised for the dry condition of the rock mass and thus is not applicable to the weak rock mass with the RMR below 18.

#### Residual value of GSI

The guideline for the GSI was presented in^25^, which are to characterise the peak strength parameters of the EPP rock mass. Considering the strain-softening effect, Reference^27^ extended the GSI framework to consider the residual strength. In their study, through the in-situ block shear test at a number of real construction sites, the residual value of the GSI, denoted as GSI^r^, was expressed with a function of the peak value of GSI, denoted as GSI^p^:

9 $${\text{GSI}}^{{\text{r}}} = {\text{GSI}}^{{\text{p}}} \cdot e^{{ - 0.0134{\text{GSI}}^{{\text{p}}} }}$$

Here, GSI^p^ varies between 25 and 75 with 5 even intervals to consider the rock mass from very poor to excellent qualities. GSI^r^ is calculated by substituting GSI^p^ into Eq. (9) with values of GSI^p^ and GSI^r^ listed in Table 1.

**Table 1:** GSI^p^ and GSI^r^.

GSI^p^	25	30	35	40	45	50	55	60	65	70	75
GSI^r^	17.9	20.1	21.9	23.4	24.6	25.6	26.3	26.9	27.2	27.4	27.5

## Geological parameters

### Within the plastic softening area

The parameters $m_{b}$, $s$, and $a$ for the SS rock mass can be calculated as^2^:

10 $$\omega (\eta ) = \left\{ \begin{aligned} & \omega^{{\text{p}}} - (\omega^{{\text{p}}} - \omega^{{\text{r}}} )\frac{\eta }{{\eta^{*} }},{0 < }\eta < \eta^{*} \hfill \\ & \omega^{{\text{r}}} ,\eta \ge \eta^{*} \hfill \\ \end{aligned} \right.$$

where $\omega$ represents any of $m_{b}$, $s$ and $a$. The peak and residual values of the strength parameters are denoted with superscripts ‘p’ and ‘r’, respectively, having $m_{b}^{{\text{p}}}$, $s^{{\text{p}}}$, $a^{{\text{p}}}$, and $m_{b}^{{\text{r}}}$, $s^{{\text{r}}}$, $a^{{\text{r}}}$. The value of $\omega$ decays linearly with the increase in $\eta$ when the rock mass is undergoing plastic softening, while it keeps unchanged with the value of $\eta$ above the critical value $\eta^{ * }$. $\omega$ equates to $\omega^{{\text{p}}}$ within the EPP rock mass and is $\omega^{r}$ within the plastic area of the EBP rock mass. The deformation modulus $E_{{\text{r}}}$ and strength parameters, such as $\sigma_{{{\text{ci}}}}$ and $m_{{\text{i}}}$, also need to be determined. A number of compression tests show that $E_{{\text{r}}}$ deteriorates for the rock mass beyond the peak state^28,29^. It is proposed that $\sigma_{{{\text{ci}}}}$ wanes from its peak value to the residual during the softening stage since the rock mass quality is weakened, and the variations of $E_{{\text{r}}}$ and $\sigma_{{{\text{ci}}}}$ also obey Eq. (10)^4^. Therefore, $E_{{\text{r}}}$, $\sigma_{{{\text{ci}}}}$, and $m_{{\text{i}}}$ within the plastic softening area are all assumed to obey Eq. (10).

As observed in Eq. (10), the prerequisite for obtaining $E_{{\text{r}}}$, $\sigma_{{{\text{ci}}}}$, $m_{{\text{i}}}$, $m_{{\text{b}}}$, $s$ and $a$ in the softening area is to predict the peak and residual values ($E_{{\text{r}}}^{{\text{p}}}$, $E_{{\text{r}}}^{{\text{r}}}$, $\sigma_{{{\text{ci}}}}^{{\text{p}}}$, $\sigma_{{{\text{ci}}}}^{{\text{r}}}$, $m_{{\text{i}}}^{{\text{p}}}$, $m_{{\text{i}}}^{{\text{r}}}$, $m_{{\text{b}}}^{{\text{p}}}$,$m_{{\text{b}}}^{{\text{r}}}$, $s^{{\text{p}}}$, $s^{{\text{r}}}$, $a^{{\text{p}}}$, $a^{{\text{r}}}$). Based on GSI^p^ and GSI^r^, the derivation of $E_{{\text{r}}}^{{\text{p}}}$, $E_{{\text{r}}}^{{\text{r}}}$, $\sigma_{{{\text{ci}}}}^{{\text{p}}}$, $\sigma_{{{\text{ci}}}}^{{\text{r}}}$, $m_{{\text{i}}}^{{\text{p}}}$, $m_{{\text{i}}}^{{\text{r}}}$, $m_{{\text{b}}}^{{\text{p}}}$,$m_{{\text{b}}}^{{\text{r}}}$, $s^{{\text{p}}}$, $s^{{\text{r}}}$, $a^{{\text{p}}}$, $a^{{\text{r}}}$ is presented in the following.

### Within the plastic elastic and plastic residual areas

#### Deformation modulus $E_{{\text{r}}}$

Empirical equations to determine *E*_r_ were proposed with GSI and RMR.

Reference^7^:

11 $$E_{{\text{r}}} = 2{\text{RMR}} - 100$$

Reference^30^:

12 $$E_{{\text{r}}} = 10^{{{{\left( {{\text{RMR}} - 10} \right)} \mathord{\left/ {\vphantom {{\left( {{\text{RMR}} - 10} \right)} {40}}} \right. \kern-\nulldelimiterspace} {40}}}}$$

Reference^31^:

13 $$E_{{\text{r}}} = 0.1\left( {\frac{{{\text{RMR}}}}{10}} \right)^{3}$$

Simplified Hoek and Diederichs equation^32^:

14 $$E_{{\text{r}}} = 100\left( {\frac{{1 - {D \mathord{\left/ {\vphantom {D 2}} \right. \kern-\nulldelimiterspace} 2}}}{{1 + e^{{\left( {{{\left( {75 + 25D - GSI} \right)} \mathord{\left/ {\vphantom {{\left( {75 + 25D - GSI} \right)} {11}}} \right. \kern-\nulldelimiterspace} {11}}} \right)}} }}} \right)$$

With GSI^p^ and GSI^r^ listed in Table 1, the calculated $E_{{\text{r}}}^{{\text{p}}}$ and $E_{{\text{r}}}^{{\text{r}}}$ from Eqs. (11)–(14) are shown in Table 2. In Table 3, *E*_r_^p^ and *E*_r_^r^ can be estimated as the average values from Eqs. (11)–(14).

**Table 2:** Calculated values of *E*_r_^p^ and *E*_r_^r^ by Eqs. (11) to (14).

GSI^p^	Equation (11)	Equation (12)	Equation (13)	Equation (14)	GSI^r^	Equation (11)	Equation (12)	Equation (13)	Equation (14)
25		3.162	2.700	1.050	17.883		2.099	1.198	0.953
30		4.217	4.288	1.645	20.069		2.381	1.575	1.126
35		5.623	6.400	2.567	21.897		2.645	1.946	1.295
40		7.499	9.113	3.986	23.403		2.885	2.291	1.452
45		10.000	12.500	6.138	24.623		3.094	2.599	1.592
50	10	13.335	16.638	9.341	25.585		3.271	2.861	1.712
55	20	17.783	21.600	13.965	26.320		3.412	3.072	1.809
60	30	23.714	27.463	20.365	26.852		3.518	3.232	1.883
65	40	31.623	34.300	28.719	27.205		3.590	3.340	1.934
70	50	42.170	42.188	38.828	27.399		3.631	3.401	1.962
75	60	56.234	51.200	50.000	27.453		3.642	3.418	1.970

**Table 3:** Estimated values of *E*_r_^p^ and *E*_r_^r^.

GSI^p^	*E*_r_^p^ (MPa)	GSI^r^	*E*_r_^r^(MPa)
75	54.359	27.453	3.010
70	43.296	27.399	2.998
65	33.660	27.205	2.955
60	25.385	26.852	2.878
55	18.337	26.320	2.764
50	12.328	25.585	2.615
45	7.160	24.623	2.429
40	5.149	23.403	2.209
35	3.648	21.897	1.962
30	2.537	20.069	1.694
25	1.728	17.883	1.417

#### Strength constant m_i_

In the previous works, such as Reference^33–35^, $m_{{\text{i}}}$ was approximated by two methods. One is to determine the classification of $m_{{\text{i}}}$ from the rock type, such as in Hoek and Brown^34^. The other method is to estimate $m_{{\text{i}}}$ from the rock mass quality. Although the latter method tends to be subjective, it enables to establish a direct relationship between $m_{{\text{i}}}$ and the rock mass strength classification^14^. Therefore, the latter method is utilised in this study to correlate $m_{{\text{i}}}$ with GSI. The test data of $m_{{\text{i}}}$ for different GSI by Hoek and Brown^33^ and Hoek and Marinos^36^ is listed in Table 4. The data for estimating $m_{{\text{i}}}$ by GSI can be best-fitted by,

15 $$m_{{\text{i}}} = 0.7375{\text{GSI}}^{0.7586}$$

**Table 4:** Values of *m*_i_ with different GSI: (a) Hoek and Brown^33^; (b) Hoek and Marino^36^.

(a) GSI	75	50	30	75	75	65	20	24
*m*_i_	25	12	8	16.3	17.7	15.6	9.6	10

(b) GSI	20	5	13	28				
*m*_i_	8.0	2.0	5.0	11.0				

**Figure 2:**
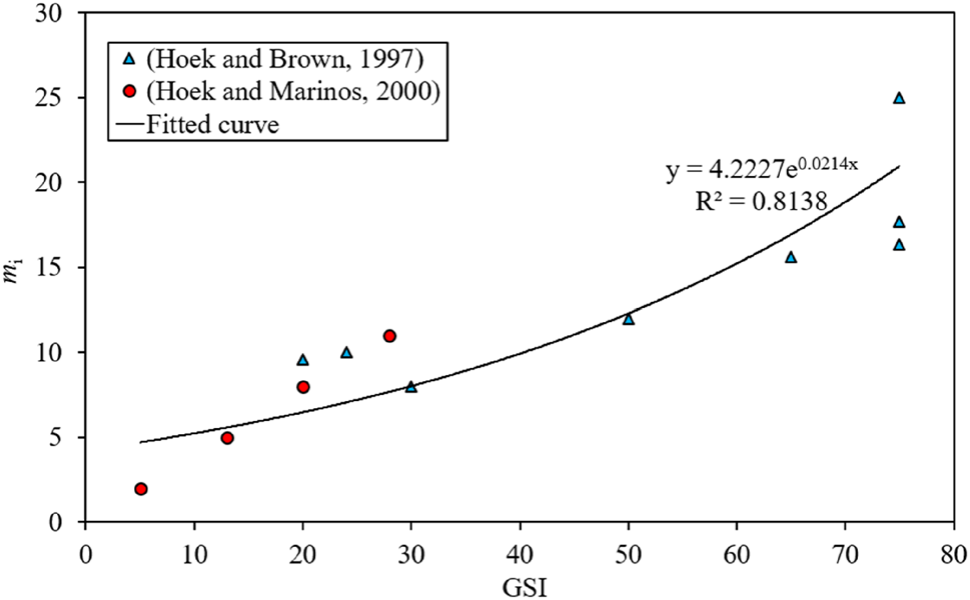
Fitting for *m*_i_.

The coefficient of determination *R*^2^ reaches 81.38%, which indicates that the fitting line is in agreement with the test results. By Eq. (15) (see Fig. 2), the calculated *m*_i_^p^ and *m*_i_^r^ with different GSI^p^ and GSI^r^ are presented in Table 5.

**Table 5:** Estimated values of *m*_i_^p^ and *m*_i_^r^.

GSI^p^	*m*_i_^p^	GSI^r^	*m*_i_^r^
75	19.507	27.453	9.101
70	18.512	27.399	9.087
65	17.500	27.205	9.038
60	16.469	26.852	8.949
55	15.417	26.320	8.814
50	14.342	25.585	8.627
45	13.240	24.623	8.380
40	12.108	23.403	8.063
35	10.942	21.897	7.666
30	9.734	20.069	7.176
25	8.477	17.883	6.575
20	7.157	15.298	5.840

It is admitted that *m*_i_ is the inherent characteristic of the intact rock. In this respect, *m*_i_ corresponds to GSI = 100. But from many references^33–36^, it is found that generally a greater GSI gives rise to a larger value of *m*_i_. Hence, in the analysis, a rough and immature relation between GSI and *m*_i_ is proposed as shown in Eq. (15) is proposed. The aim of Eq. (15) is to solve the tunnel strain as one of the input parameter in the latter. And according to Eq. (15), the tunnel strain is greater in comparison to a constant *m*_i_ with no reduction. Then, the tunnel design will be conservative and safe. In this respect, Eq. (15) is reasonable. Furthermore, the sensitive analysis for the influence of multiple mechanical parameters on the tunnel strain has also been undertaken. It is found that in comparing with other input parameters such as the deformation modulus and the compressive rock strength, the effect of *m*_i_ on the rock deformation is trivial. In this aspect, although Eq. (15) is subjective, it seems to be not very important factor that affect the results in this analysis.

#### Strength constants m_b_, s and a

According to Eqs. (5) to (7), when the disturbance factor *D* is 0, $m_{{\text{b}}}^{{\text{p}}}$ and $m_{{\text{b}}}^{{\text{r}}}$ can be obtained from GSI^p^, GSI^r^, $m_{{\text{i}}}^{{\text{p}}}$, and $m_{{\text{i}}}^{{\text{r}}}$; and $s^{{\text{p}}}$, $s^{{\text{r}}}$, $a^{{\text{p}}}$, $a^{{\text{r}}}$ can be calculated from GSI^p^ and GSI^r^. The estimated result is listed in Table 6.

**Table 6:** Estimated values of *m*_b_^p^, *s*^p^, *a*^p^ and *m*_b_^r^, *s*^r^, *a*^r^.

GSI^p^	*m*_b_^p^	*s*^p^	*a*^p^	GSI^r^	*m*_b_^r^	*s*^r^	*a*^r^
75	7.988	62.177	0.501	27.453	0.682	0.316	0.527
70	6.341	35.674	0.501	27.399	0.680	0.314	0.527
65	5.014	20.468	0.502	27.205	0.671	0.307	0.527
60	3.947	11.744	0.503	26.852	0.656	0.295	0.528
55	3.090	6.738	0.504	26.320	0.634	0.278	0.529
50	2.405	3.866	0.506	25.585	0.605	0.257	0.530
45	1.857	2.218	0.508	24.623	0.568	0.230	0.532
40	1.421	1.273	0.511	23.403	0.523	0.201	0.535
35	1.074	0.730	0.516	21.897	0.471	0.170	0.539
30	0.799	0.419	0.522	20.069	0.413	0.139	0.544
25	0.582	0.240	0.531	17.883	0.350	0.109	0.550
20	0.411	0.138	0.544	15.298	0.284	0.082	0.560

#### Compressive strength of intact rock σ_ci_

Here, *σ*_ci_ by GSI is calculated in three steps.

1. Estimation of *σ*_cm_/*σ*_ci_
  Considering different RMR, the reduction factor *σ*_cm_/*σ*_ci_ was proposed by Wilson^37^ to characterise the rock mass strength decreasing from its peak value to the residual. Assuming RMR-5 equals to GSI (see Eq. (8)), the estimated *σ*_cm_/*σ*_ci_ by Asef et al.^14^ are listed in Table 7. Other fitting equations for *σ*_cm_/*σ*_ci_ in the literature are presented in Eqs. (16) to (22):

**Table 7:** Estimated values of *σ*_cm_/*σ*_ci_ proposed by Asef et al.^14^.

RMR	20	30	40	50	60	70	80	90	100
*σ*_cm_/*σ*_ci_	0.147	0.142	0.142	0.166	0.200	0.250	0.400	0.666	1.000
  Reference^34^:
  16 $$\frac{{\sigma_{{{\text{cm}}}} }}{{\sigma_{{{\text{ci}}}} }} = \sqrt {e^{{\left( {\frac{{{\text{RMR}} - {100}}}{9}} \right)}} }$$
  Reference^38^:
  17 $$\frac{{\sigma_{{{\text{cm}}}} }}{{\sigma_{{{\text{ci}}}} }} = e^{{\left( {0.0765{\text{RMR}} - 7.65} \right)}}$$
  Reference^39^:
  18 $$\frac{{\sigma_{{{\text{cm}}}} }}{{\sigma_{{{\text{ci}}}} }} = e^{{\left( {\frac{{{\text{RMR}} - 100}}{24}} \right)}}$$
  Reference^40^:
  19 $$\frac{{\sigma_{{{\text{cm}}}} }}{{\sigma_{{{\text{ci}}}} }} = e^{{\left( {\frac{{{\text{RMR}} - 100}}{20}} \right)}}$$
  Reference^41^:
  20 $$\frac{{\sigma_{{{\text{cm}}}} }}{{\sigma_{{{\text{ci}}}} }} = e^{{\left( {\frac{{{\text{RMR}} - 100}}{18.75}} \right)}}$$
  Reference^42^:
  21 $$\frac{{\sigma_{{{\text{cm}}}} }}{{\sigma_{{{\text{ci}}}} }} = \frac{{{\text{RMR}}}}{{{\text{RMR}} + 6\left( {100 - {\text{RMR}}} \right)}}$$
  Reference^26^:
  22 $$\frac{{\sigma_{{{\text{cm}}}} }}{{\sigma_{{{\text{ci}}}} }} = 0.019e^{{0.05{\text{GSI}}}} ,5 \le {\text{GSI}} \le 35$$
  The GSI was given values from 5 to 95 with 10 intervals, which is to compute *σ*_cm_/*σ*_ci_ through Eqs. (16) to (22). The otained *σ*_cm_/*σ*_ci_ by Eqs. (16) to (22), by Asef et al.^14^, and the field data retrieved from realistic construction sites^42^ are plotted in Fig. 3. With the estimated *σ*_cm_/*σ*_ci_, the best-fitting equation is expressed as:
  23 $$\frac{{\sigma_{{{\text{cm}}}} }}{{\sigma_{{{\text{ci}}}} }} = 0.0103e^{{0.0476{\text{GSI}}}}$$

**Figure 3:**
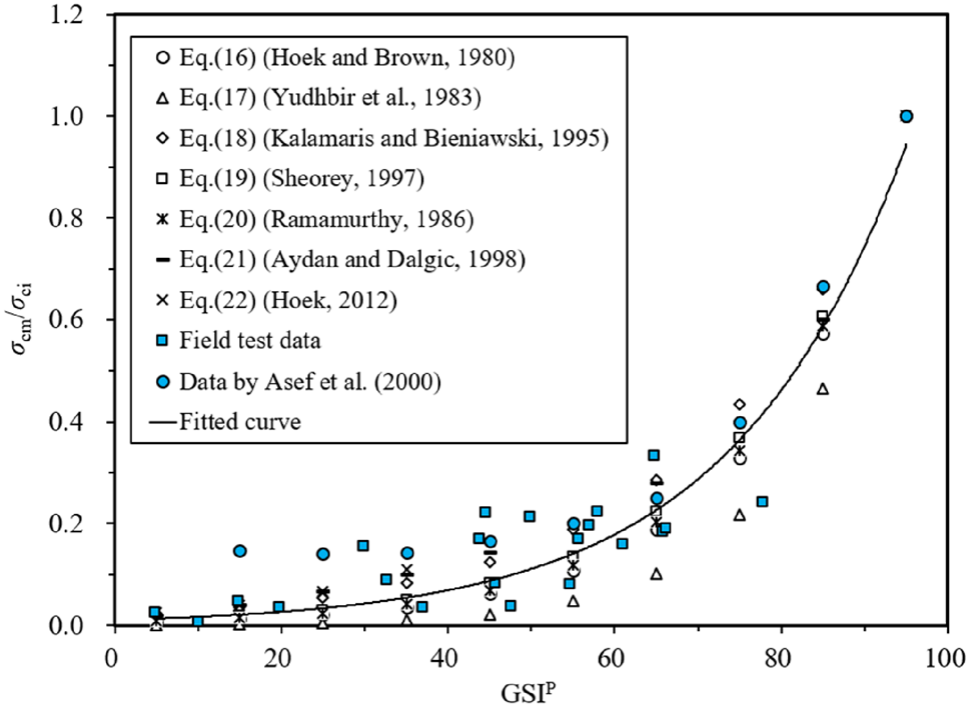
Fitting for *σ*_ci_/*σ*_cm_.
  The coefficient of determination *R*^2^ is 95.84%, which indicates the prediction by Eq. (23) is acceptable.
2. Estimation of *σ*_cm_ and *σ*_ci_.
  Reference^43^ claimed that *σ*_cm_ can be described as a function of RMR:
  24 $$\sigma_{{{\text{cm}}}} = 0.5e^{{0.06{\text{RMR}}}}$$
  Combing Eqs. (23) and (24), the solution for *σ*_ci_ is derived as:
  25 $$\sigma_{{{\text{ci}}}} = \frac{{0.5e^{{0.06{\text{RMR}}}} }}{{0.0387 + 0.00474e^{{\frac{{{\text{GSI}}}}{18.9086}}} }}$$
  *σ*_ci_^p^ and *σ*_ci_^r^ with different values of GSI^p^ and GSI^r^ are calculated by Eq. (25), and the result is presented in Table 8.

**Table 8:** Estimated values of *σ*_ci_^p^ and *σ*_ci_^r^.

GSI^p^	*σ*_ci_^p^ (MPa)	GSI^r^	*σ*_ci_^r^ (MPa)
75	237.222	27.4	66.781
70	219.950	27.4	66.629
65	201.942	27.2	66.088
60	183.276	26.9	65.111
55	164.134	26.3	63.656
50	144.813	25.6	61.682
45	125.708	24.6	59.160
40	107.271	23.4	56.066
35	89.957	21.9	52.406
30	74.155	20.1	48.201

## Semi-analytical procedure

### Governing equation

For the case of plane strain, the equilibrium equation is:

26 $$\frac{{\partial \sigma_{{\text{r}}} }}{\partial r} + \frac{{\sigma_{{\text{r}}} - \sigma_{{\uptheta }} }}{r} = 0$$

In terms of small strain case, the displacement compatibility is:

27 $$\varepsilon_{{\text{r}}} = \frac{du}{{dr}},\;\;\varepsilon_{{\uptheta }} = \frac{u}{r}$$

### Stresses and strains in the plastic softening zone

The generalised H-B failure criterion^33^ is :

28 $$\sigma_{1} = \sigma_{3} + \sigma_{{{\text{ci}}}} \left( {m_{{\text{b}}} \sigma_{3} /\sigma_{{{\text{ci}}}} + s} \right)^{a}$$

where $\sigma_{1}$ and $\sigma_{3}$ are the major and minor principal stresses. $\sigma_{{{\text{ci}}}}$ is the uniaxial compression strength of intact rock. $m_{{\text{b}}}$ and $s$ are the strength constants, respectively. According to Eq. (28), the yielding function of the rock mass surrounding a circular opening is:

29 $$f(\sigma_{{\uptheta }} ,\sigma_{{\text{r}}} ,\eta ) = \sigma_{{\uptheta }} - \sigma_{{\text{r}}} - \sigma_{{{\text{ci}}}} (\eta )\left[ {m_{{\text{b}}} (\eta )\sigma_{{\text{r}}} /\sigma_{{{\text{ci}}}} + s(\eta )} \right]^{a(\eta )}$$

First, $\sigma_{{{\text{r}}2}}$, the radial stress at the elastic–plastic boundary is solved by combing Eq. (26) with Eq. (29) through Runge–Kutta method.

A constant radial stress increment is assumed for each annulus, i.e.:

30 $$\Delta \sigma_{{\text{r}}} = \sigma_{{{\text{r}}(i)}} - \sigma_{{{\text{r}}(i - 1)}}$$

where $\sigma_{{{\text{r}}(i)}}$ and $\sigma_{{{\text{r}}(i - 1)}}$ are the radial stresses at the inner and outer boundaries of each annulus (i.e. *r* = *r*_(*i*)_ and *r*_(*i*-1)_).

The plastic strain is expressed as:

31 $$\left\{ {\begin{array}{*{20}l} {\varepsilon _{{{\text{r}}(i)}} } \hfill \\ {\varepsilon _{{1\uptheta (i)}} } \hfill \\ \end{array} } \right\} = \left\{ {\begin{array}{*{20}l} {\varepsilon _{{{\text{r}}(i - 1)}} } \hfill \\ {\varepsilon _{{\uptheta (i - 1)}} } \hfill \\ \end{array} } \right\} + \left\{ {\begin{array}{*{20}l} {\Delta \varepsilon _{{{\text{r}}(i)}}^{{{\text{elas}}}} } \hfill \\ {\Delta \varepsilon _{{\uptheta (i)}}^{{{\text{elas}}}} } \hfill \\ \end{array} } \right\} + \left\{ {\begin{array}{*{20}l} {\Delta \varepsilon _{{{\text{r}}(i)}}^{{{\text{plas}}}} } \hfill \\ {\Delta \varepsilon _{{\uptheta (i)}}^{{{\text{plas}}}}} \hfill \\ \end{array} } \right\}$$

where $\varepsilon_{{\text{r}(i)}}$ and $\varepsilon_{{{\uptheta }}_{(i)}}$ are the radial and tangential strains at *r* = *r*_(*i*)_; $\varepsilon_{{{\text{r}}(i - 1)}}$ and $\varepsilon_{{{\uptheta }}_{(i - 1)}}$ are the radial and tangential strains at *r* = *r*_(*i*-1)_; $\Delta \varepsilon_{{{\text{r}}(i)}}^{{{\text{plas}}}}$ and $\Delta \varepsilon_{{{\uptheta }}_{(i)}}^{{{\text{plas}}}}$ are the radial and tangential plastic strain increments; $\Delta \varepsilon_{{{{\text{r}}(i)}}}^{{{\text{elas}}}}$ and $\Delta \varepsilon_{{{\uptheta }}_{(i)}}^{{{\text{elas}}}}$ are the radial and tangential elastic strain increments.

According to Hooke’s law, the elastic strain increments can be correlated to the stress increments, i.e.:

32 $$\left\{ \begin{gathered} \Delta \varepsilon_{{{\text{r}}(i)}}^{{{\text{elas}}}} \hfill \\ \Delta \varepsilon_{{{\uptheta }(i)}}^{{{\text{elas}}}} \hfill \\ \end{gathered} \right\} = \frac{1 + \mu }{E}\left[ {\begin{array}{*{20}c} {1 - \mu } \\ { - \mu } \\ \end{array} } \right.\left. {\begin{array}{*{20}c} { - \mu } \\ {1 - \mu } \\ \end{array} } \right]\left\{ \begin{gathered} \Delta \sigma_{{{\text{r}}(i)}} \hfill \\ \Delta \sigma_{{{\uptheta }}_{(i)}} \hfill \\ \end{gathered} \right\}$$

The relation between $\varepsilon_{{\uptheta }}^{{{\text{plas}}}}$ and $\varepsilon_{{\text{r}}}^{{{\text{plas}}}}$ can be given as:

33 $$\varepsilon_{{\text{r}}}^{{{\text{plas}}}} = - K_{\psi } (\eta )\varepsilon_{{\uptheta }}^{{{\text{plas}}}}$$

where $K_{\psi } (\eta )$ is the dilatancy coefficient and can be written as:

34 $$K_{\psi } (\eta ) = \frac{1 + \sin \psi (\eta )}{{1 - \sin \psi (\eta )}}$$

where $\psi$ is the dilatancy angle, it should be noted that $\psi$ is not equal to the friction angle $\varphi$ when the non-associated flow rule is employed.

In order to solve the strain components, Eq. (27) can be rewritten as:

35 $$\varepsilon_{{{\text{r}}(i)}} = \frac{{\Delta u_{(i)} }}{{\Delta r_{(i)} }},\;\varepsilon_{{{\uptheta }}_{(i)}} = \frac{{u_{(i)} }}{{r_{(i)} }}$$

where $u_{(i)}$ is the radial displacement at *r* = *r*_(*i*)_; substituting Eqs. (32) and (33) into Eqs. (31) and (36), one gains:

36 $$\varepsilon _{{{{\uptheta (i)}}}} = \frac{{u_{{(i)}} }}{{r_{{(i)}} }} = \frac{{A_{{(i - 1)}} (r_{{(i)}} /r_{{(i - 1)}} - 1) + u_{{(i - 1)}} /r_{{(i - 1)}} }}{{r_{{(i)}} /r_{{(i - 1)}} + K_{{\psi (i)}} (r_{{(i)}} /r_{{(i - 1)}} - 1)}}$$

37 $$\varepsilon _{{{\text{r}}(i)}} = \frac{{\Delta u_{{(i)}} }}{{\Delta r_{{(i)}} }} = - K_{{\psi (i)}} \varepsilon _{{{{\uptheta (i)}}}} + A_{{(i - 1)}} \cdot \frac{{r_{{(i)}} /r_{{(i - 1)}} - 1}}{{1 - r_{{(i - 1)}} /r_{{(i)}} }}$$

38 $$u_{(i)} = \frac{{A_{(i - 1)} r_{(i)} (r_{(i)} - r_{(i - 1)} ) + u_{(i - 1)} r_{(i)} }}{{r_{(i)} + K_{\psi } (r_{(i)} - r_{(i - 1)} )}}$$

where

$$A_{(i - 1)} = \frac{(1 + \nu )}{E}\left\{ {\Delta \sigma_{{{\text{r}}(i)}} (1 - \nu - K_{\psi } \nu ) + \left[ { - \sigma_{{{\uptheta }}_{(i - 1)}} + \sigma_{{{\text{r}}(i)}} + H(\sigma_{{{\text{r}}(i)}} ,\eta_{(i - 1)} )} \right](K_{\psi } - K_{\psi } \nu - \nu )} \right\} + \varepsilon_{{{\text{r}}(i - 1)}} + K_{\psi } \varepsilon_{{{\uptheta }}_{(i - 1)}}$$

$$H(\sigma_{{{\text{r}}(i)}} ,\eta_{(i - 1)} ) = \sigma_{{{\text{ci}}}} \left( {m_{{{\text{b}}(i - 1)}} \sigma_{{{\text{r}}(i)}} /\sigma_{{{\text{ci}}}} + s_{(i - 1)} } \right)^{a\left( i \right)}$$

In accordance with Reference^4^, the relation between $r_{(i)}$ and $r_{(i - 1)}$ can be derived as:

39 $$\frac{{r_{(i)} }}{{r_{(i - 1)} }} = \frac{{2H\left[ {(\sigma_{{{\text{r}}(i)}} + \sigma_{{{\text{r}}(i - 1)}} )/2,\eta_{(i - 1)} } \right] + \Delta \sigma_{{\text{r}}} }}{{2H\left[ {(\sigma_{{{\text{r}}(i)}} + \sigma_{{{\text{r}}(i - 1)}} )/2,\eta_{(i - 1)} } \right] - \Delta \sigma_{{\text{r}}} }}$$

As illustrated in Eqs. (36), (37) and (39), $\varepsilon_{{{\uptheta }}_{(i)}}$,$\varepsilon_{{{\text{r}}(i)}}$ and $r_{(i)} /r_{(i - 1)}$ are independent of the radius $R_{{\text{p}}}$, or $R_{{\text{r}}}$. This means that with no need to obtain the value of $R_{{\text{r}}}$, stress and strain components in the plastic softening zone can be solved first.

### Radii of plastic softening and residual zones

IN the plastic residual zone, by incorporating Eq. (29) into Eq. (26), one obtains:

40 $$\frac{{\partial \sigma_{{\text{r}}} }}{\partial r} = \frac{{\sigma_{{{\text{ci}}}} \left( {m_{{\text{b}}}^{{\text{r}}} \sigma_{{\text{r}}} /\sigma_{{{\text{ci}}}}^{{\text{r}}} + s^{{\text{r}}} } \right)^{{a^{{\text{r}}} }} }}{r}$$

where $m_{{\text{b}}}^{{\text{r}}}$ and *s*^r^ are the strength parameters in the residual zone. The boundary conditions for Eq. (41) are: (1) *r* = *R*_0_, *σ*_r_ = *p*_i_; and (2) *r* = *R*_r_, *σ*_r_ = *σ*_r1_. Hence, the following equation can be derived from Eq. (40):

41 $$R_{{\text{r}}} = R_{0} \exp \left[ {\frac{{\left( {\sigma_{{{\text{r}}1}} m_{{\text{b}}}^{{\text{r}}} /\sigma_{{{\text{ci}}}}^{{\text{r}}} + s^{{\text{r}}} } \right)^{{a^{{\text{r}}} }} - \left( {p_{i} m_{{\text{b}}}^{{\text{r}}} /\sigma_{{{\text{ci}}}}^{{\text{r}}} + s^{{\text{r}}} } \right)^{{a^{{\text{r}}} }} }}{{m_{{\text{b}}}^{{\text{r}}} (1 - a^{{\text{r}}} )}}} \right]$$

Equation (41) illustrates that $R_{{\text{r}}}$ can be obtained by use of $m_{{\text{b}}}^{{\text{r}}}$, $s^{{\text{r}}}$ and $\sigma_{{{\text{r}}1}}$. In fact, from Eq. (40), the relation between $R_{{\text{r}}}$ and $R_{{\text{p}}}$ can be derived as follows:

42 $$\frac{{R_{{\text{r}}} }}{{R_{{\text{p}}} }} = \prod\limits_{i = 1}^{j} {\frac{{2H(\sigma_{{{\text{r}}(i)}}^{\prime } ,\eta_{(i - 1)} ) + \Delta \sigma_{{\text{r}}} }}{{2H(\sigma_{{{\text{r}}(i)}}^{\prime } ,\eta_{(i - 1)} ) - \Delta \sigma_{{\text{r}}} }}}$$

where *j* is the number of the annulus immediately outside the plastic softening-residual boundary. Equation (42) shows that $R_{{\text{p}}}$ can be solved by $R_{{\text{r}}}$.

In addition, when only the plastic softening zone is formed, Eq. (42) can be rewritten into:

43 $$\frac{{R_{{\text{p}}} }}{{R_{0} }} = \prod\limits_{i = 1}^{j} {\frac{{2H(\sigma_{{{\text{r}}(i)}}^{\prime } ,\eta_{(i - 1)} ) + \Delta \sigma_{{\text{r}}} }}{{2H(\sigma_{{{\text{r}}(i)}}^{\prime } ,\eta_{(i - 1)} ) - \Delta \sigma_{{\text{r}}} }}}$$

### Radial displacement of plastic softening and residual zones

Essentially, after obtaining $R_{{\text{p}}}$, $u_{(i)}$ in the plastic softening zone can be solved by Eq. (38). As for *u* in the plastic residual zone, it can be obtained in a closed-form as shown in Eq. (44)^44^. Since the plastic softening zone is considered herein, $R_{{\text{r}}}$ and $\sigma_{{{\text{r}}1}}$ of Eq. (44) are substituted for $R_{{\text{p}}}$ and $\sigma_{0}$ of Eq. (38) presented in the elastic-brittle-plastic solution^44^.

44 $$\frac{u}{r} = \frac{1}{2G}\frac{1}{{r^{{K_{\psi } }} }}\left[ {D_{1} f_{1} (r) + D_{2} f_{2} (r) + D_{3} f_{3} (r) + 2R_{{\text{r}}}^{{K_{\psi } }} Gu|_{{r = R_{{\text{r}}} }} - D_{1} f_{1} (r) - D_{2} f_{2} (r) - D_{3} f_{3} (r)} \right]$$

where *G* is the shear modulus, *G* = *E*/2(1 + *μ*).

$$A^{{H - B}} = \left( {m_{{\text{b}}}^{{\text{r}}} \sigma _{{{\text{ci}}}}^{{\text{r}}} p_{{\text{i}}} + s^{{\text{r}}} \sigma _{{{\text{ci}}}}^{{{\text{r2}}}} } \right)^{{a_{{\text{r}}} }} ,B^{{H - B}} = m_{{\text{b}}}^{{\text{r}}} \sigma _{{{\text{ci}}}}^{{\text{r}}} /4$$

$$D_{1} = (K_{\psi } - \mu K_{\psi } - \mu )A^{H - B} + (K_{\psi } + 1)(1 - 2\mu )(p_{{\text{i}}} - \sigma_{{{\text{r}}{1}}} ),$$

$$D_{2} = (K_{\psi } + 1)(1 - 2\mu )A^{H - B} + 2(K_{\psi } - \mu K_{\psi } - \mu )B^{H - B} ,D_{3} = (K_{\psi } + 1)(1 - 2\mu )B^{H - B} ,$$

$$f_{1} (r) = r^{{K_{\psi } + 1}} /(K_{\psi } + 1),f_{2} (r) = \frac{{r^{{K_{\psi } + 1}} }}{{(K_{\psi } + 1)}}\left[ {\ln \left( {\frac{r}{{R_{0} }}} \right) - \frac{1}{{K_{\psi } + 1}}} \right]$$

$$f_{3} (r) = \frac{{r^{{K_{\psi } + 1}} }}{{(K_{\psi } + 1)}}\left[ {\ln^{2} \left( {\frac{r}{{R_{0} }}} \right) - \frac{2}{{K_{\psi } + 1}}\ln \left( {\frac{r}{{R_{0} }}} \right) + \frac{2}{{(K_{\psi } + 1)^{2} }}} \right].$$

## Verification

**Figure 4:**
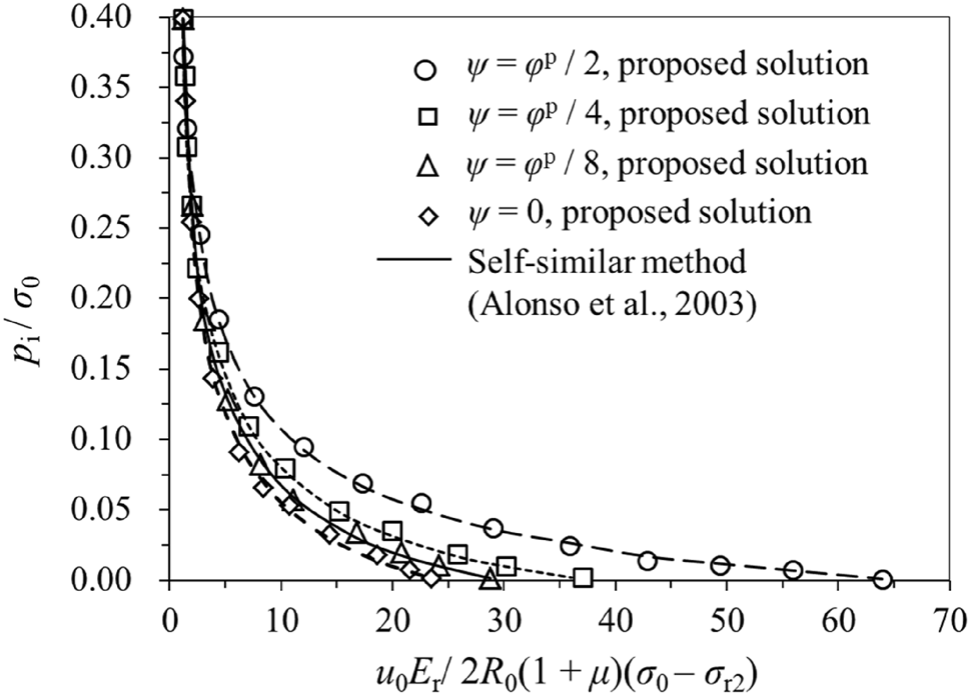
Variations of dimensionless support pressure *p*_i_/*σ*_0_ versus dimensionless radial displacement *u*_0_*E*_r_/ 2*R*_0_(1 + *μ*)(*σ*_0_ – *σ*_r2_) for case C1, C2, C3 and C4.

**Figure 5:**
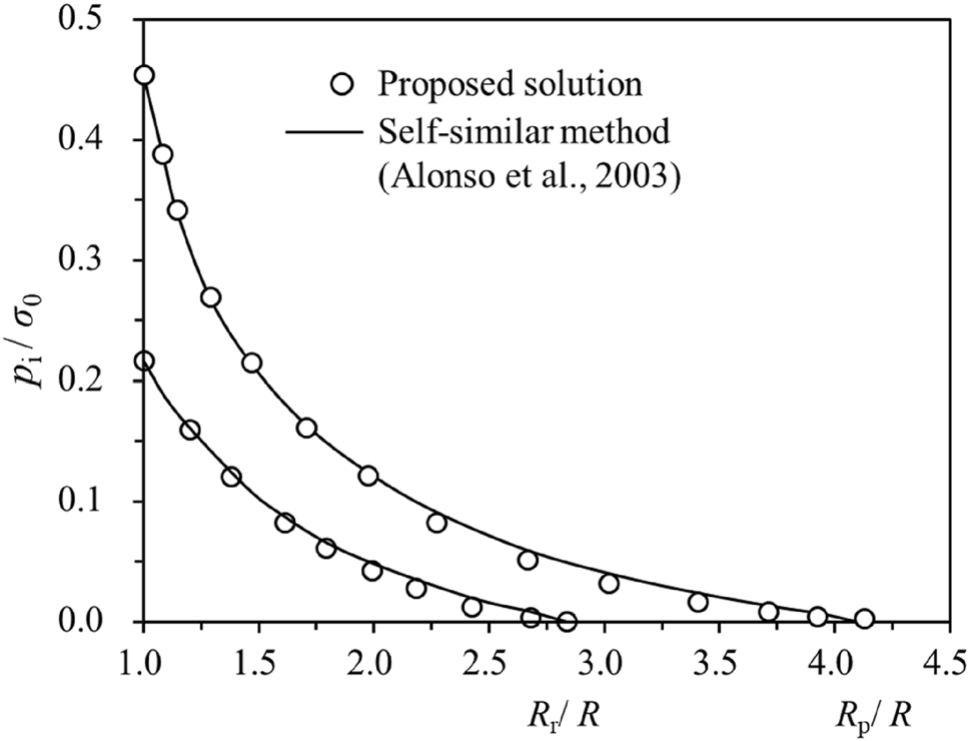
Variations of dimensionless support pressure *p*_i_/*σ*_0_ versus plastic radius *R*_r_/*R*_0_, or *R*_p_/*R*_0_ for case C5.

**Figure 6:**
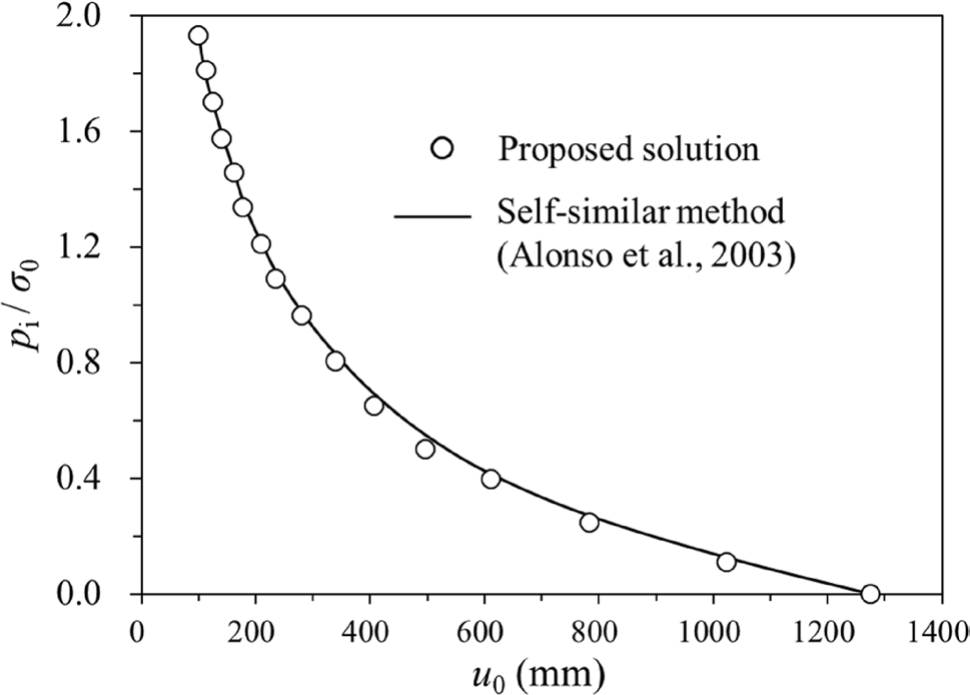
Ground reaction curve for case C6.

The strength parameters for a group of tunnel excavation cases are used to verify the proposed semi-analytical procedure (Table 9). The cases are from the References^2,3,6^. Figure 4 demonstrates the distribution of the normalised radial displacement predicted by the semi-analytical procedure and a self-similar method^2^ for the SS rock masses with different dilatancy behaviours, which show good agreement with each other. The normalised support pressure versus the normalised plastic radii is plotted in Fig. 5. Comparison of the Ground Reaction Curves for the SS rock mass obtained from the semi-analytical procedure and self-similar method^2^ are presented in Fig. 6, also showing good convergence. Therefore, the semi-analytical procedure proposed in this study is sufficiently reliable in predicting the tunnel strain for the SS rock masses. It should be noted that EPP and EPB rock masses can be regarded as the special cases for the SS rock masses. In this aspect, the above can also serve as a verification of the EPP and EPB rock masses.

**Table 9:** Parameters of the tunnel cases for verification.

	C1	C2	C3	C4	C5	C6
*ψ*/°	*φ*^p^/2	*φ*^p^/4	*φ*^p^/8	0	3.75	3.1
*φ*p/°	30	30	30	30	30	24.81
*φ*r/°	22	22	22	22	22	15.69
η*	0.008	0.008	0.008	0.008	0.008	0.017
*E*_r_/GPa	10	10	10	10	3.837	3.837
$\mu$	0.25	0.25	0.25	0.25	0.25	0.25
*R*_0_/m	3	3	3	3	3	7
$c^{{\text{p}}} /{\text{MPa}}$	1	1	1	1	1	0.744
$c^{{\text{r}}} /{\text{MPa}}$	0.7	0.7	0.7	0.7	0.7	0.397
$\sigma_{0} /{\text{MPa}}$	20	20	20	20	20	12

In some existing studies, efforts were given to calculate the tunnel strain *ɛ*_θ_ for the EPP rock mass with a wide range of qualities^13–16^. Particularly, Reference^16^ established a regression model with 20 < RMR < 90:

45 $$u_{0} \left( {{\text{mm}}} \right) = \left\{ \begin{aligned} & 24711 \times 0.43^{{p_{{\text{i}}} }} \times {\text{RMR}}^{ - 2.42} ,\sigma_{0} = 2.7 \; {\text{MPa}} \hfill \\ & 157513 \times 0.59^{{p_{{\text{i}}} }} \times {\text{RMR}}^{ - 2.71} ,\sigma_{0} = 5.4 \; {\text{MPa}} \hfill \\ & 696395 \times 0.65^{{p_{{\text{i}}} }} \times {\text{RMR}}^{ - 2.99} ,\sigma_{0} = 8.1 \; {\text{MPa}} \hfill \\ & 3973329 \times 0.66^{{p_{{\text{i}}} }} \times {\text{RMR}}^{ - 3.37} ,\sigma_{0} = 10.8 \; {\text{MPa}} \hfill \\ & 18531047 \times 0.67^{{p_{{\text{i}}} }} \times {\text{RMR}}^{ - 3.72} ,\sigma_{0} = 13.5\; {\text{MPa}} \hfill \\ \end{aligned} \right.$$

Based on Eqs. (8), (45) can be transferred to the following equation,

46 $$u_{0} \left( {{\text{mm}}} \right) = \left\{ \begin{aligned} & 24711 \times 0.43^{{p_{{\text{i}}} }} \times \left( {{\text{GSI}} + 5} \right)^{ - 2.42} ,\sigma_{0} = 2.7\; {\text{MPa}} \hfill \\ & 157513 \times 0.59^{{p_{{\text{i}}} }} \times ({\text{GSI}} + 5)^{ - 2.71} ,\sigma_{0} = 5.4 \; {\text{MPa}} \hfill \\ & 696395 \times 0.65^{{p_{{\text{i}}} }} \times {\text{(GSI + 5)}}^{ - 2.99} ,\sigma_{0} = 8.1 \; {\text{MPa}} \hfill \\ & 3973329 \times 0.66^{{p_{{\text{i}}} }} \times {\text{(GSI + 5)}}^{ - 3.37} ,\sigma_{0} = 10.8 \; {\text{MPa}} \hfill \\ & 18531047 \times 0.67^{{p_{{\text{i}}} }} \times {\text{(GSI + 5)}}^{ - 3.72} ,\sigma_{0} = 13.5 \; {\text{MPa}} \hfill \\ \end{aligned} \right.$$

The value of *ε*_θ_ predicted by the proposed procedures in this study can be compared with that by Eq. (46).

**Figure 7:**
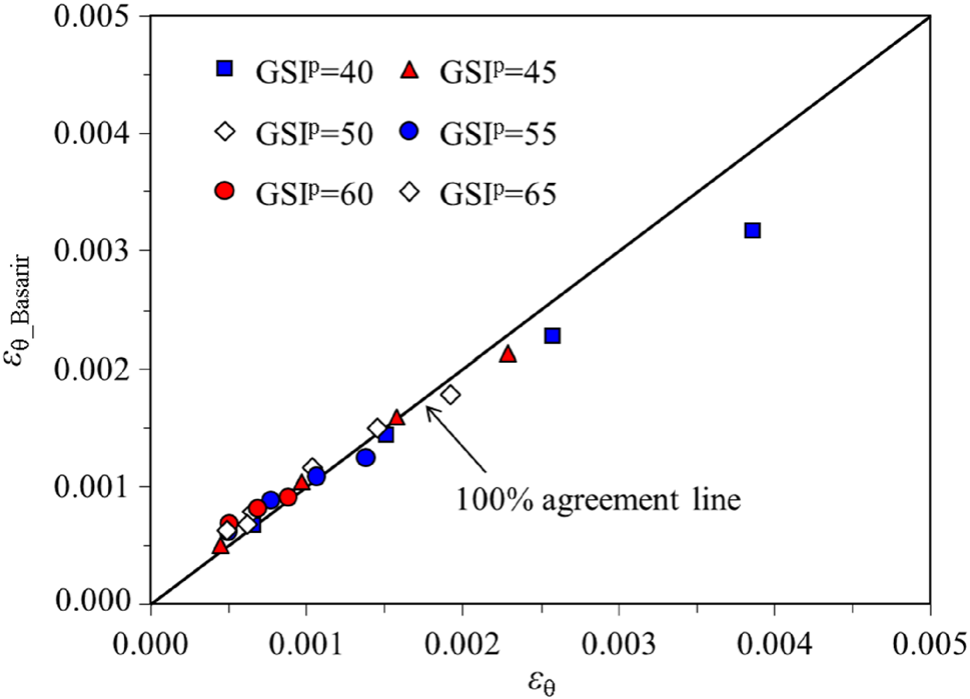
Comparison between *ε*_θ_ and *ε*_θ_Basarir_.

Then GSI^p^ was varied between 40 and 65 with 5 intervals to compare the proposed method with that by Reference^16^. For each GSI^p^ value, *σ*_0_ ranges from 2.7 to 13.5 MPa, *p*_i_ is 0 and *R*_0_ is 5 m. *ε*_θ_Basarir_ is obtained by dividing *u*_0_ by *R*_0_^16^. The comparison of *ε*_θ_ obtained from the semi-analytical procedure and *ε*_θ_basarir_ by the scheme in Reference^16^ shows good agreement with the coefficient of determination *R*^2^ up to 90.8% (see Fig. 7). Then the rationality of the input geological parameters (*E*_r_^p^, *E*_r_^r^, *σ*_ci_^p^, *σ*_ci_^r^, *m*_i_^p^, *m*_i_^r^, *m*_b_^p^*, m*_b_^r^*,s*^p^*, s*^r^*, a*^p^*,* and *a*^r^) in this study can be validated to some extent.

## Regression model for tunnel strain

The strain *ε*_θ_ can be fitted as a function of GSI^p^, *σ*_0_ and *p*_i_/*σ*_0_ by a nonlinear regression method. The equations for *ε*_θ_ in the plastic and elastic areas differ from each other:

47a $$\varepsilon_{{\uptheta }} = f_{1} \left( {{\text{GSI}}^{{\text{p}}} ,\sigma_{0} ,{{p_{{\text{i}}} } \mathord{\left/ {\vphantom {{p_{{\text{i}}} } {\sigma_{0} }}} \right. \kern-\nulldelimiterspace} {\sigma_{0} }}} \right),\varepsilon_{{\uptheta }} > \varepsilon_{{{\uptheta }2}} ,p_{{\text{i}}} < \sigma_{{{\text{r2}}}} ,{\text{ plastic}}\;{\text{area}}$$

47b $$\varepsilon_{{\uptheta }} = f_{2} \left( {{\text{GSI}}^{{\text{p}}} ,\sigma_{0} ,{{p_{{\text{i}}} } \mathord{\left/ {\vphantom {{p_{{\text{i}}} } {\sigma_{0} }}} \right. \kern-\nulldelimiterspace} {\sigma_{0} }}} \right),\varepsilon_{{\uptheta }} \le \varepsilon_{{{\uptheta }2}} ,p_{{\text{i}}} \ge \sigma_{{{\text{r2}}}} ,\;{\text{elastic area}}$$

In Eq. (38), the critical strain *ε*_θ2_ and the critical support pressure *σ*_r2_ need to be determined prior to solving *ε*_θ_. Combining Eqs. (26) and (27), fitting equations for *σ*_r2_ and *ε*_θ2_ can be written as:

48a $$\sigma_{{{\text{r2}}}} = f_{3} \left( {{\text{GSI}}^{{\text{p}}} ,\sigma_{0} } \right)$$

48b $$\varepsilon_{{{\theta 2}}} = f_{4} \left( {{\text{GSI}}^{{\text{p}}} ,\sigma_{0} } \right)$$

The Taylor series polynomial regression (PR) can be adopted to solve *f*_1_, *f*_3_ and *f*_4_. Particularly for *f*_1_, a nonlinear function can be constructed as:

49 $$\begin{aligned} y & = \exp \left( {a_{1} + b_{1} x_{1} + b_{2} x_{2} + b_{3} x_{3} + c_{1} x_{1}^{2} + c_{2} x_{2}^{2} + c_{3} x_{3}^{2} + c_{4} x_{1} x_{2} + c_{5} x_{3} x_{2} + c_{6} x_{3} x_{1} } \right. \hfill \\ & \quad + d_{1} x_{3}^{2} x_{2} + d_{2} x_{3}^{2} x_{1} + d_{3} x_{3} x_{1}^{2} + d_{4} x_{3} x_{2}^{2} + d_{5} x_{1} x_{2} x_{3} + d_{6} x_{2}^{3} + d_{7} x_{1}^{3} \hfill \\ & \quad + e_{1} x_{1}^{2} x_{2}^{2} + e_{2} x_{2}^{2} x_{3}^{2} + e_{3} x_{3}^{2} x_{1} x_{2} + e_{4} x_{3} x_{1}^{3} + e_{5} x_{3} x_{2}^{3} \hfill \\ & \quad \left. { + f_{1} x_{1}^{3} x_{3}^{2} + f_{2} x_{2}^{3} x_{3}^{2} } \right) \hfill \\ \end{aligned}$$

For *f*_3_ and *f*_4_, the variable *y* (*ε*_θ2_ or *σ*_r2_) depends on *x*_1_ (GSI^p^) and *x*_2_ (*σ*_0_), as:

50 $$y = a_{1} + b_{1} x_{1} + b_{2} x_{2} + c_{1} x_{1}^{2} + c_{2} x_{2}^{2} + c_{3} x_{1} x_{2} + d_{1} x_{1}^{3} + d_{2} x_{2}^{3} + d_{3} x_{1} x_{2}^{2} + d_{4} x_{2} x_{1}^{2}$$

As for *f*_2_, the relation between the variable *y* (*ε*_θ_) and the independent variables *x*_1_ (GSI^p^), *x*_2_ (*σ*_0_), and *x*_3_ (*p*_i_/*σ*_0_) can be derived from Eq. (28) as:

51 $$y = \frac{{x_{2} \left( {1 + \mu } \right)\left( {1 - x_{3} } \right)}}{{a_{1} x_{1}^{3} + b_{1} x_{1}^{2} + c_{1} x_{1} + d_{1} }}$$

To obtain the coefficients in Eqs. (40) to (42), *ε*_θ_ for a large number of tunnelling cases are calculated by the proposed iterative procedure. The input geological parameters (GSI^p^, GSI^r^, *E*_r_^p^, *E*_r_^r^, *σ*_ci_^p^, *σ*_ci_^r^, *m*_b_^p^*, m*_b_^r^*, s*^p^*, s*^r^*, a*^p^*,* and *a*^r^) for the calculation are given in Tables 1, 3, 6, and 8. Nine values for η^*^ within cases A1 to A9 are listed in Table 10. *σ*_0_ varies from 5 to 50 MPa with intervals of 5 MPa. *p*_i_/*σ*_0_ ranges from 0 to 1 MPa and 10 to 20 values are selected for different combination of *p*_i_ and *σ*_r2_. *f*_2_, *f*_3_ and *f*_4_ are merely correlated to the peak geological parameters in the elastic zone. The regression model is composed of twelve equations: three equations are for *f*_2_, *f*_3_ and *f*_4_, and nine equations are for *f*_1_. Then the coefficients can be determined with the Levenberg Marquardt iteration algorithm (see Tables 11 and 12), which is validated through the analysis of variance ANOVA. The predictions with the twelve equations match well with those by the semi-analytical procedure.

**Table 10:** Critical plastic softening parameter η^*^.

Case	A1	A2	A3	A4	A5	A6	A7	A8	A9
η*	0	0.005	0.01	0.025	0.05	0.1	0.5	1	∞

**Table 11:** Coefficients in *f*_4_, *f*_3_, and *f*_2_.

	*f*_4_		*f*_3_		*f*_2_
*a*_1_ (10^–5^)	42.5845	*a*_1_	− 0.28589	*a*_1_	0.000273
*b*_1_ (10^–5^)	2.88088	*b*_1_	0.0338	*b*_1_	− 0.01925
*b*_2_ (10^–5^)	1.1807	*b*_2_	0.91007	*c*_1_	0.47982
*c*_1_ (10^–5^)	0.075412	*c*_1_	− 0.00195	*d*_1_	− 3.57994
*c*_2_ (10^–5^)	0.043128	*c*_2_	0.00938		
*c*_3_ (10^–5^)	0.035988	*c*_3_	− 0.01975		
*d*_1_ (10^–5^)	0.000485	*d*_1_ (10^–5^)	2.44631		
*d*_2_ (10^–5^)	0.000479	*d*_2_ (10^–5^)	− 7.68786		
*d*_3_ (10^–5^)	0.000468	*d*_3_ (10^–5^)	1.3393		
*d*_4_ (10^–5^)	0.000488	*d*_4_ (10^–5^)	5.4923

**Table 12:** Coefficients in *f*_1_: (a) when η^*^ = ∞, 1, 0.5, 0.1, 0.05; (b) when η^*^ = 0.025, 0.01, 0.005, 0.

(a) η*	∞	1	0.5	0.1	0.05
*a*_1_	0.37576	− 0.74117	− 0.61639	1.08615	3.27504
*b*_1_	− 0.3432	− 0.24502	− 0.2742	− 0.43415	− 0.58165
*b*_2_	− 16.0768	− 16.14255	− 16.55177	− 16.45784	− 19.47774
*b*_3_	0.32959	0.41235	0.46672	0.53739	0.53157
*c*_1_	0.00788	0.00246	0.00225	0.00656	0.00967
*c*_2_	19.49741	21.77123	22.57404	21.46441	27.19204
*c*_3_	− 0.00289	− 0.00176	− 0.00156	− 0.00362	− 0.00485
*c*_4_	0.33925	0.34786	0.36874	0.38357	0.45668
*c*_5_	− 0.07987	− 0.22695	− 0.26771	− 0.70855	− 0.82604
*c*_6_	− 0.00862	− 0.02094	− 0.02172	− 0.01372	− 0.00686
*d*_1_	− 0.00059963	− 0.01182	− 0.00343	0.00441	0.00765
*d*_2_ (10^–5^)	2.24755	0.707777	− 0.862592	4.88805	10.3286
*d*_3_ (10^–5^)	4.96784	15.5397	17.8538	8.07328	− 8.19822
*d*_4_	− 0.01828	0.29111	0.37644	0.83708	0.87071
*d*_5_	− 0.00656	− 0.02268	− 0.00153	0.00261	0.00278
*d*_6_	− 15.49841	− 18.33127	− 18.88734	− 19.65351	− 25.08435
*d*_7_ (10^–5^)	− 1.72834	− 0.615086	− 0.667556	− 3.36496	− 5.69299
*e*_1_ (10^–4^)	− 7.9539	− 29.9	− 33.9	− 21.9	− 36.7
*e*_2_ (10^–4^)	14.6	23.1	16.2	− 76.6	− 95.5
*e*_3_ (10^–5^)	9.11563	8.73084	10.4683	1.56558	− 2.26792
*e*_4_ (10^–7^)	− 2.61481	− 9.51432	− 11.4793	− 0.354321	12.6512
*e*_5_	0.06741	− 0.27619	− 0.2907	− 0.48179	− 0.34204
*f*_1_ (10^–9^)	0.532421	1.95193	3.18653	− 3.039	− 8.42
*f*_2_	− 0.00215	− 0.00076716	0.00017157	0.00582	0.00517

(b) η*	0.025	0.01	0.005	0	
*a*_1_	3.37806	− 1.23767	− 0.45896	3.37629	
*b*_1_	− 0.56164	− 0.16611	− 0.15858	− 0.47128	
*b*_2_	− 21.86517	− 21.45617	− 19.50907	− 16.89839	
*b*_3_	0.48318	0.52196	0.38419	0.25426	
*c*_1_	0.0088	− 0.00075226	− 0.00132	0.00759	
*c*_2_	33.78561	37.16971	26.33782	28.57907	
*c*_3_	− 0.00656	− 0.00627	− 0.00162	− 0.00173	
*c*_4_	0.51334	0.43875	0.29709	0.15241	
*c*_5_	− 0.96887	− 0.66871	− 0.00379	0.38674	
*c*_6_	− 0.00277	− 0.00527	− 0.0063	0.00259	
*d*_1_	0.01421	0.00686	− 0.0018	− 0.00575	
*d*_2_ (10^–5^)	14.2824	10.6465	− 4.79792	− 2.97141	
*d*_3_ (10^–5^)	− 19.9824	2.54789	19.5277	− 4.91271	
*d*_4_	0.85951	0.57685	− 0.19027	− 0.13331	
*d*_5_	0.0037	0.00075557	− 0.01386	− 0.02578	
*d*_6_	− 30.75648	− 32.48237	− 20.64928	− 36.72157	
*d*_7_ (10^–5^)	− 4.78882	2.51696	2.35951	− 5.6103	
*e*_1_ (10^–4^)	− 68.6	− 81.9	25.7	109.2	
*e*_2_ (10^–4^)	− 97.7	− 19.3	94.2	− 39.7	
*e*_3_ (10^–5^)	− 7.26793	− 3.79109	12.6535	32.7694	
*e*_4_ (10^–7^)	19.6994	− 4.32378	− 20.7783	4.66801	
*e*_5_	− 0.18899	− 0.15045	0.17124	0.25735	
*f*_1_ (10^–9^)	− 10.6367	− 2.62886	13.3079	2.50107	
*f*_2_	0.00376	− 0.00163	− 0.00931	0.00429	

## Parametric study

### Variation of tunnel strain with different critical softening parameters

**Figure 8:**
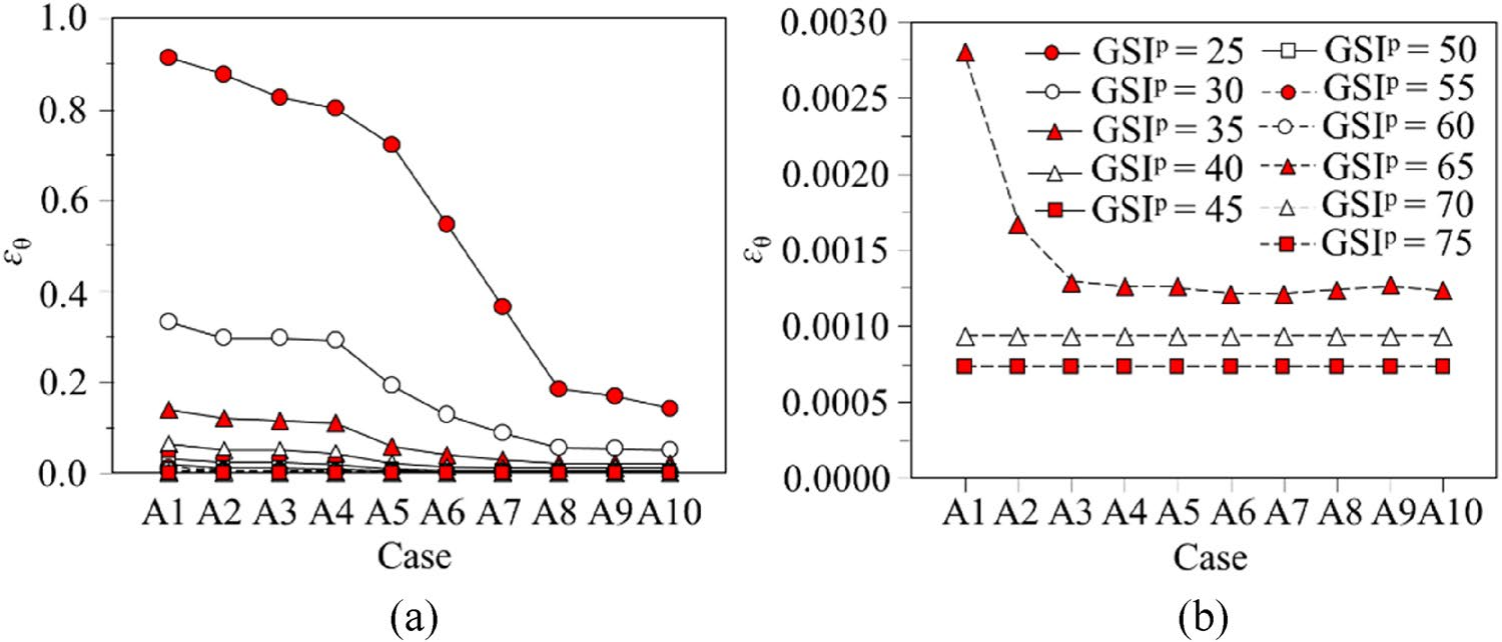
Variation of *ε*_θ_ versus cases A1 to A9: (**a**) GSI^p^ ranges from 25 to 75; (**b**) GSI^p^ = 65, 70, 75.

**Figure 9:**
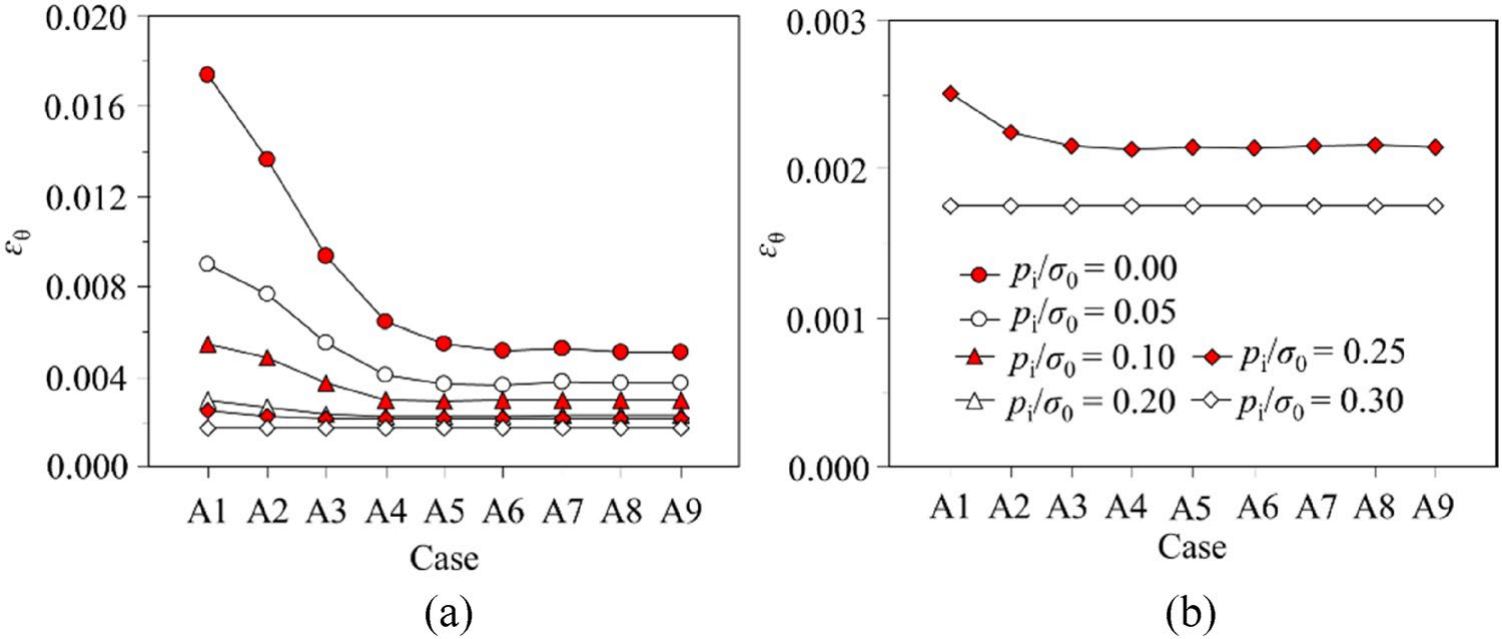
Variation of *ε*_θ_ versus cases A1 to A9: (**a**) *p*_i_/*σ*_0_ ranges from 0 to 0.3; (**b**) *p*_i_/*σ*_0_ = 0.25, *p*_i_/*σ*_0_ = 0.30.

Values of *ε*_θ_ are calculated by the proposed regression model, which are plotted for Cases A1 to A9 versus GSI^p^, *σ*_0_, and *p*_i_/*σ*_0_, respectively, as in Figs. 8 and 9. In Fig. 8, GSI^p^ is variable, *σ*_0_ is 30 MPa and *p*_i_/*σ*_0_ is 0.1, and in Fig. 9, *p*_i_/*σ*_0_ is variable, GSI^p^ is 30 and *σ*_0_ is 5 MPa. When GSI^p^ is 70 or 75, and *p*_i_/*σ*_0_ is 0.3, *ε*_θ_ maintains constant. The reason is that GSI^p^ and *p*_i_/*σ*_0_ are relatively large, so that the rock mass takes elastic deformations and is independent of η^*^. With plastic deformations in the rock mass, *ε*_θ_ decreases to a substantial constant with the increase in η^*^. The decrease of *ε*_θ_ is induced by the shrinkage of the plastic residual area. If η^*^ is nil, all rock mass within the plastic area is characterised with the residual strength; and the maximum *ε*_θ_ is therefore reached; as η^*^ increases, *ε*_θ_ falls rapidly since the softening area expands; and *ε*_θ_ becomes stable when the softening zone dominates in the plastic area. The expansion of the plastic residual area is the critical factor enhancing the deformation within the rock mass. In the practical engineering, the measures to decrease the plastic residual zone can substantially improve the tunnel stability. Furthermore, *ε*_θ_ falls quickly and becomes constant within a small η^*^ for excellent quality rock mass, whereas *ε*_θ_ for the weak rock mass decreases slowly and the decline process is prolonged until a plateau is reached (see Fig. 9). Hence, the rock mass deformation decreases more suddenly with a better quality rock while η^*^ increases.

### Difference of tunnel strain between the EPP and EBP rock masses

**Figure 10:**
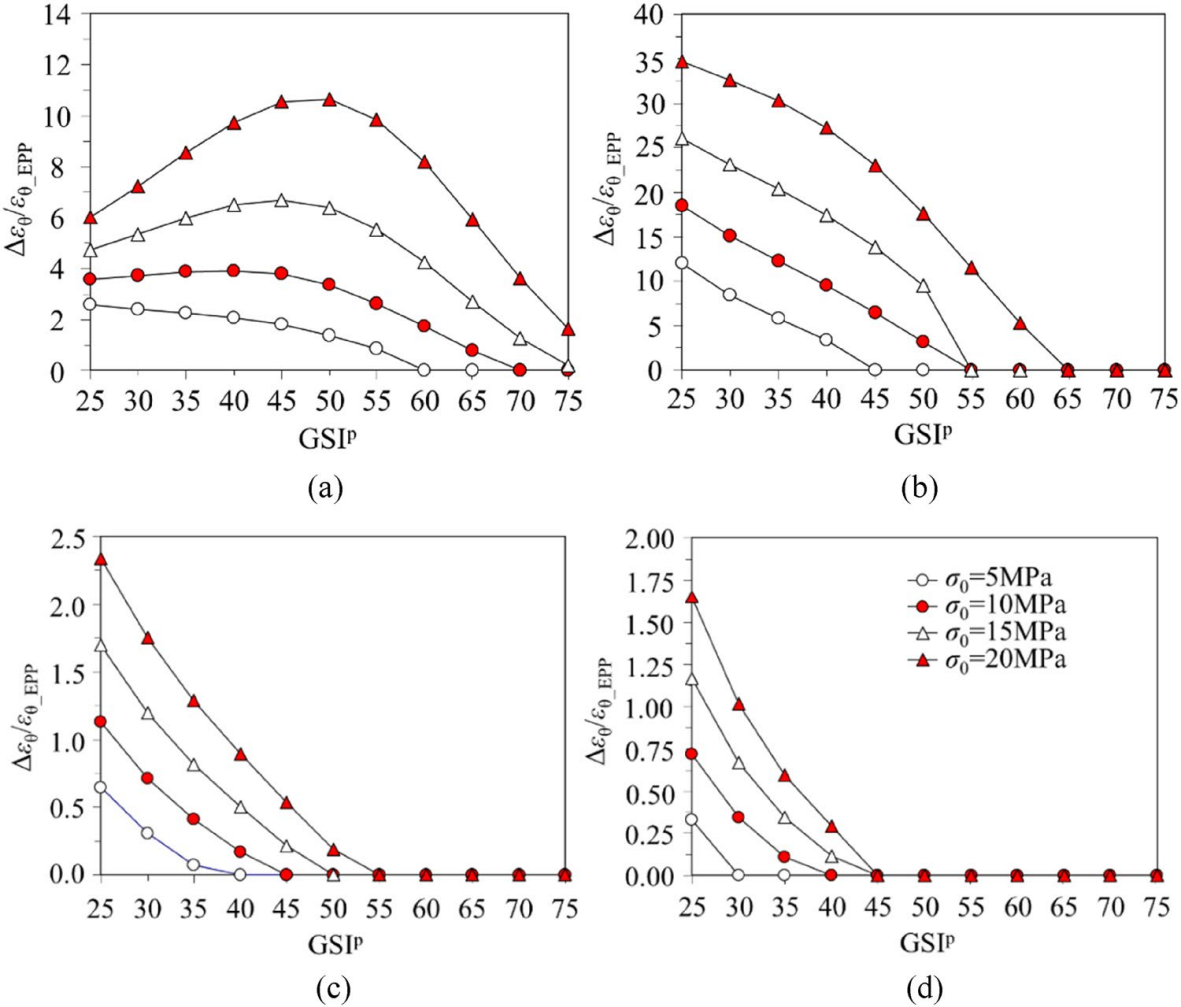
Variation of Δ*ε*_θ_/*ε*_θ_EPP_ versus GSI^p^ : (**a**) *p*_i_/*σ*_0_ = 0; (**b**) *p*_i_/*σ*_0_ = 0.1; (**c**) *p*_i_/*σ*_0_ = 0.2; (**d**) *p*_i_/*σ*_0_ = 0.3.

*ε*_θ_ for the EPP rock mass is symbolised by *ε*_θ_EPP_. The increase ratio of *ε*_θ_ for the EBP rock mass in comparison to the EPP counterpart is denoted by Δ*ε*_θ_/*ε*_θ_EPP_. Δ*ε*_θ_/*ε*_θ_EPP_ versus GSI^p^ for variations in *σ*_0_ and *p*_i_/*σ*_0_ is plotted in Fig. 10.

When *p*_i_/*σ*_0_ is 0.1, 0.2 and 0.3, Δ*ε*_θ_/*ε*_θ_EPP_ decreases as GSI^p^ increases (see Fig. 10b–d). Hence, while *p*_i_/*σ*_0_ exceeds 0.1, the effect of η^*^ on *ε*_θ_ for the weakest rock mass (GSI^p^ = 25) is the greatest, which should be highlighted. While *p*_i_ is 0, and *σ*_0_ ranges from 10 to 20 MPa, Δ*ε*_θ_/*ε*_θ_EPP_ rises but then decreases with the increase in GSI^p^ (Fig. 10a). The maximum Δ*ε*_θ_/*ε*_θ_EPP_ appears while GSI^p^ is around 45 or 50. In this case, the influence of η^*^ on *ε*_θ_ for the moderate rock mass (GSI^p^ = 45, 50) is the largest. For GSI^p^ is 50 and *σ*_0_ is 20 MPa, Δ*ε*_θ_/*ε*_θ_EPP_ reaches almost 10.64 for *p*_i_/*σ*_0_ is 0 but drops to 1.77 for *p*_i_/*σ*_0_ is 0.1 (see Fig. 10a,b). This means that the growth of *p*_i_ effectively weakens the softening effect on the deformation for moderate quality rock mass with high initial stress. Furthermore, when GSI^p^ is greater than 55 and *p*_i_/*σ*_0_ exceeds 0.1, Δ*ε*_θ_/*ε*_θ_EPP_ for most cases is 0, which means *ε*_θ_ by EPP and EBP rock masses are equivalent (see Fig. 10b–d). This is because that the rock mass undergoes an elastic deformation. Therefore, if *p*_i_/*σ*_0_ reaches 0.1, the rock mass deformation is inconsiderable and irrespective of η^*^ for the excellent rock mass quality (GSI^p^ ≥ 55).

### Sensitive analysis

**Figure 11:**
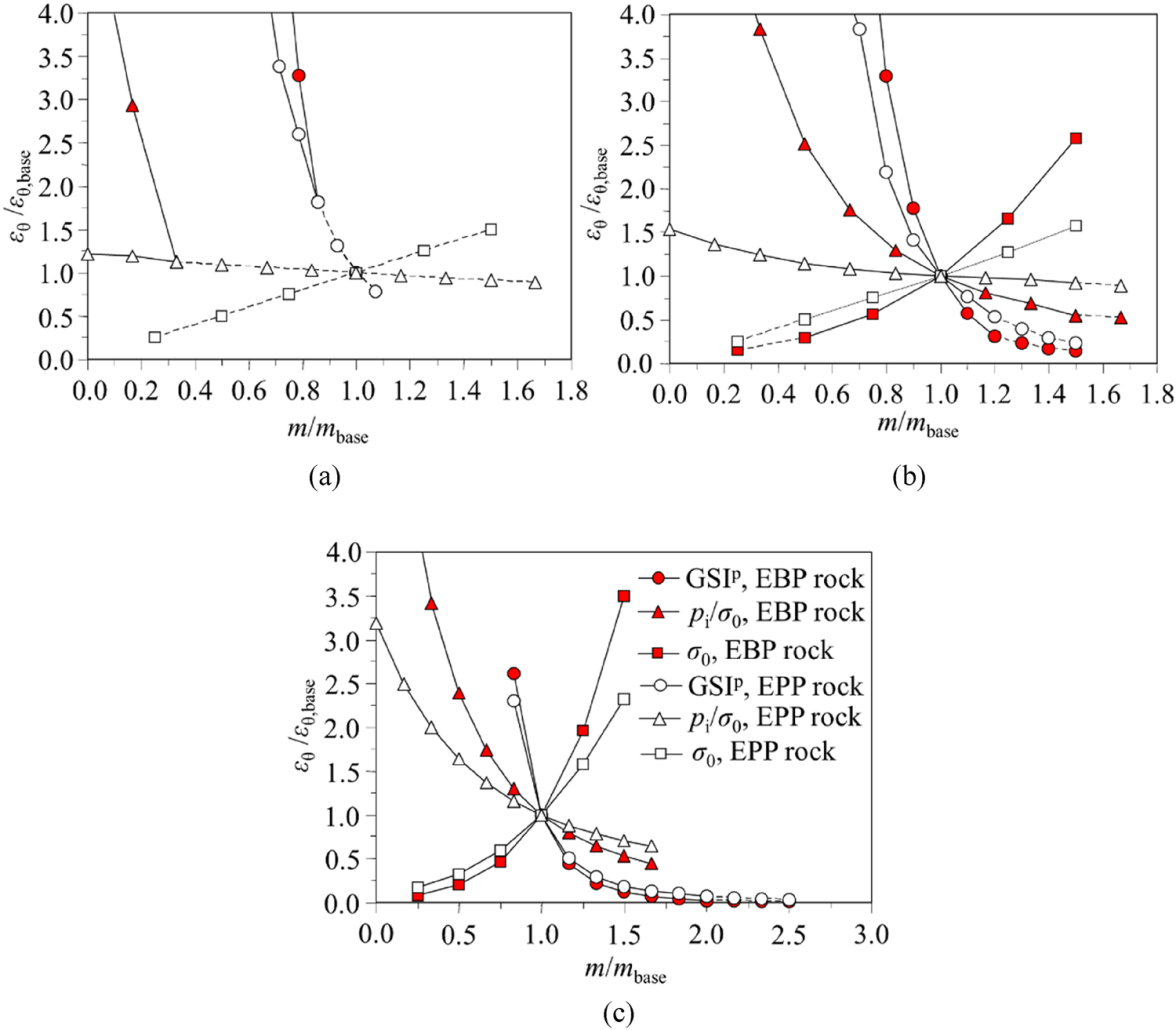
Sensitive analysis of GSI^p^, *σ*_0_ and *p*_i_/*σ*_0_ on *ε*_θ_: (**a**) cases B1; (**b**) case B2; (**c**) case B3.

Figure 11 illustrates the sensitivity analysis concerning the tunnel strain *ɛ*_θ_, showing the relative significance of the most significant input data (i.e. GSI^p^, *σ*_0_ and *p*_i_/*σ*_0_) on this final output (i.e. *ɛ*_θ_). Three base cases with different rock mass qualities are given in Table 13. In the sensitive analysis, *σ*_0_ varies between 5 and 30 MPa with even intervals of 5 MPa. *p*_i_/*σ*_0_ ranges from 0 to 0.225 with 0.025 intervals. GSI^p^ ranges from 25 to 75 with 5 intervals. GSI^p^, *σ*_0_ or *p*_i_/*σ*_0_ is represented by the variable *m*. GSI^p^, *σ*_0_ or *p*_i_/*σ*_0_ in cases B1 to B3 is represented by *m*_base_*. ɛ*_θ_ calculated by cases B1 to B3 is represented by *ɛ*_θ,base_.

**Table 13:** GSI, *σ*_0_ and *p*_i_/*σ*_0_ for cases B1 to B3.

	Case B1	Case B2	Case B3
GSI^p^	70	50	30
*σ*_0_ (MPa)	20	20	20
*p*_i_/*σ*_0_	0.15	0.15	0.15

In comparison with the EBP rock mass, *ε*_θ_/*ε*_θ,base_ of the EPP rock mass with the moderate and weak rock qualities tends to be closer to the line for *ε*_θ_/*ε*_θ,base_ is 1 (see Fig. 11b,c). In this respect, *ε*_θ_ for the EBP rock mass is more sensitive to the change in GSI^p^, *p*_i_/*σ*_0_ and *σ*_0_. However, for the excellent quality rock mass, *ε*_θ_/*ε*_θ,base_ of EBP rock mass coincides with that of EPP rock mass (Fig. 11a). This is attributed to that the rock mass exhibits the elastic behaviour, and thus *ε*_θ_ is independent of the plastic parameters. In this respect, the influence of GSI^p^, *p*_i_/*σ*_0_ or *σ*_0_ on *ε*_θ_ by EPP and EBP rock masses are equivalent.

Among the input parameters GSI^p^, *σ*_0_ and *p*_i_/*σ*_0_, the change in GSI^p^ gives rise to the greatest change in *ε*_θ_. Especially for the excellent rock mass, *ε*_θ_/*ε*_θ,base_ by GSI^p^ is considerably higher than *σ*_0_ and *p*_i_/*σ*_0_ (Fig. 11a). Therefore, GSI^p^ is of vital importance in controlling *ε*_θ_. The relative significance of *p*_i_/*σ*_0_ and *σ*_0_ varies with different conditions. For the EBP rock mass, when *p*_i_/*σ*_0_ decreases and *σ*_0_ increases with an equivalent variation, *ε*_θ_/*ε*_θ,base_ affected by *p*_i_/*σ*_0_ is always higher than that by *σ*_0_; and it becomes remarkably higher while *p*_i_/*σ*_0_ decreases to a small value. Hence, for the EBP rock mass, when *p*_i_/*σ*_0_ decreases and *σ*_0_ increases, the influence of *p*_i_/*σ*_0_ on *ε*_θ_ is larger than that of *σ*_0_. For all the other conditions, the influence of *σ*_0_ on *ε*_θ_ is greater than that of *p*_i_/*σ*_0_. For instance, for the EPP rock mass, the change in *σ*_0_ causes a larger variation in *ε*_θ_; for the EBP rock mass, when *p*_i_/*σ*_0_ increases and *σ*_0_ decreases with the equivalent variation, a decrease of *σ*_0_ yields a higher reduction of *ε*_θ_. As the weak rock mass shows the EPP behaviour^33^, the reduction of *σ*_0_ exerts greater influence than the increase in *p*_i_/*σ*_0_ in controlling the rock deformation for the weak rock mass. In the tunnelling engineering, the reduction of *σ*_0_ and the increase of *p*_i_/*σ*_0_ can be obtained by relieving the stress and installing the rigid support, respectively.

## Conclusions

Various GSI were considered to quantify the input geological parameters for the strain-softening rock masses with various qualities. A specialised numerical scheme was presented to calculate the tunnel strain around a circular opening within the rock mass. The proposed semi-analytical procedure and the input geological parameters were validated through comparison of the tunnel strain obtained by the semi-analytical procedure with that predicted by the previous studies. With the obtained input geological parameters, more accurate quantification of the tunnel strain was obtained by a semi-analytical procedure. A regression model, composed of 12 fitting equations, was further proposed: 3 equations were to calculate the critical tunnel strain, the critical support pressure and the tunnel strain with elastic behaviour, and 9 equations were for the tunnel strain with different strain-softening behaviours. The model provides practical guidelines to assess the deformations of the rock mass prior to the tunnel construction. Following conclusions can then be drawn:

The tunnel strain wanes to a constant value with the critical softening parameter keeps increasing, which is mainly ascribed to the shrinkage of the plastic residual area. Reversely, the rock deformation is mainly raised due to the expansion of the plastic residual area. In the practical engineering, the measures to decrease the plastic residual area can substantially improve the tunnel stability.

While the support pressure exceeds a certain value (*p*_i_/*σ*_0_ ≥ 0.1), the critical softening parameter makes the most significant influence on the tunnel strain for the weakest rock mass (GSI^p^ = 25). In comparison, with no support pressure (*p*_i_/*σ*_0_ ≥ 0) and relatively high initial stress (*σ*_0_ ≥ 10 MPa), the influence of the critical softening parameter for the moderate rock mass (GSI^p^ is around 45 or 50) is the most significant. While the support pressure that acted on the good rock mass quality (GSI^p^ ≥ 55) exceeds a certain value, the rock mass deformation becomes inconsiderable.

While the rock mass exhibits a strain-softening behaviour, the tunnel strain for the EBP rock mass can be affected by the change in the rock mass quality, the support pressure and the initial stress state. Among the three input geological parameters (i.e. GSI^p^, the support pressure, and the initial stress), GSI^p^ is of vital importance in controlling the tunnel strain. The relative significance of the support pressure and initial stress varies with different conditions. For the EBP rock mass, with the support pressure decreases and the initial stress increases, the tunnel strain is mostly influenced by the variation in the support pressure. For all other conditions, the initial stress state becomes the critical factor.

## Data Availability

The data that support the findings of this study are available from the corresponding author upon reasonable request.

## List of symbols

*η*: Softening parameter
$\sigma_{{\text{r}}}$, $\sigma_{{\uptheta }}$: Radial and tangential stresses
$\varepsilon_{{{\text{r(}}i)}}$,$\varepsilon_{{{\uptheta}}_{(i)}}$: Radial and tangential strains at *r* = *r*_(__*i*__)_
$\varepsilon_{{{\text{r}}(i - 1)}}$, $\varepsilon_{{{\uptheta }}_{(i - 1)}}$: Radial and tangential strains at *r* = *r*_(__*i*__-1)_
$\varepsilon_{{\text{r}}}^{{{\text{plas}}}}$,$\varepsilon_{{\uptheta }}^{{{\text{plas}}}}$: Radial and tangential plastic strains
$\Delta \varepsilon_{{{\text{r}}(i)}}^{{{\text{plas}}}}$,$\Delta \varepsilon_{{{\uptheta }(i)}}^{{{\text{plas}}}}$: Radial and tangential plastic strain increments
$\Delta \varepsilon_{{{\text{r}}(i)}}^{{{\text{elas}}}}$,$\Delta \varepsilon_{\theta (i)}^{{{\text{elas}}}}$: Radial and tangential elastic strain increments
$u_{(i)}$: Radial displacement at *r* = *r*_(__*i*__)_
*p*_0_: Initial ground stress
*μ*: Poisson’s ratio
*σ*_1_, *σ*_3_: Major and minor principal stresses at failure, respectively
GSI: Geological Strength Index
GSI^p^, GSI^r^: Peak and residual values of the Geological Strength Index
*ψ*: Dilatancy angle
$$K_{\psi } (\eta )$$: Dilatancy coefficient
*φ*: Friction angle
$\sigma_{{{\text{ci}}}}$: Uniaxial compression strength of intact rock
*m*_b_, *s*, *a*: Strength parameters of the Hoek–Brown rock mass
$$\omega$$: *m*_b_, *s* and *α*
*D*: Disturbance coefficient
RMR: Rock Mass Rating
*η*^*^: Critical value of the softening parameter
$\sigma_{{{\text{cm}}}}$: Strength of a rock mass
*σ*_r__(__*i*__)_,*σ*_r__(__*i*__-1)_: Radial stresses at the inner and outer boundaries of each annulus
*E*_r_^p^ , *E*_r_^r^: Peak and residual values of the deformation modulus
$\sigma_{{{\text{r}}2}}$: Radial stress at the elastic–plastic boundary
